# Population Trends and Variation in Body Mass Index from 1971 to 2008 in the Framingham Heart Study Offspring Cohort

**DOI:** 10.1371/journal.pone.0063217

**Published:** 2013-05-10

**Authors:** Jason P. Block, S. V. Subramanian, Nicholas A. Christakis, A. James O’Malley

**Affiliations:** 1 Obesity Prevention Program, Department of Population Medicine, Harvard Medical School/Harvard Pilgrim Health Care Institute, Boston, Massachusetts, United States of America; 2 Department of Social and Behavioral Sciences, Harvard School of Public Health, Boston, Massachusetts, United States of America; 3 Department of Health Care Policy, Harvard Medical School, Boston, Massachusetts, United States of America; Old Dominion University, United States of America

## Abstract

**Objective:**

We examined body mass index (BMI) across place and time to determine the pattern of BMI mean and standard deviation trajectories.

**Methods:**

We included participants in the Framingham Heart Study (FHS) Offspring Cohort over eight waves of follow-up, from 1971 to 2008. After exclusions, the final sample size was 4569 subjects with 28,625 observations. We used multi-level models to examine population means and variation at the individual and neighborhood (census tracts) levels across time with measured BMI as the outcome, controlling for individual demographics and behaviors and neighborhood poverty. Because neighborhoods accounted for limited BMI variance, we removed this level as a source of variation in final models. We examined sex-stratified models with all subjects and models stratified by sex and baseline weight classification.

**Results:**

Mean BMI increased from 24.0 kg/m^2^ at Wave 1 to 27.7 at Wave 8 for women and from 26.6 kg/m^2^ to 29.0 for men. In final models, BMI variation also increased from Waves 1 to 8, with the standard deviation increasing from 4.18 kg/m^2^ to 6.15 for women and 3.31 kg/m^2^ to 4.73 for men. BMI means increased in parallel across most baseline BMI weight classifications, except for more rapid increases through middle-age for obese women followed by declines in the last wave. BMI standard deviations also increased in parallel across baseline BMI classifications for women, with greater divergence of BMI variance for obese men compared to other weight classifications.

**Conclusion:**

Over nearly 40 years, BMI mean and variation increased in parallel across most baseline weight classifications in our sample. Individual-level characteristics, especially baseline BMI, were the primary factors in rising BMI. These findings have important implications not only for understanding the sources of the obesity epidemic in the United States but also for the targeting of interventions to address the epidemic.

## Introduction

The obesity epidemic has progressed rapidly in the United States over the last several decades. The mean body mass index (BMI) of US adults has increased from 25.7 kg/m^2^ to 28.7 for men and 25.1 kg/m^2^ to 28.7 for women from the 1960s to 2000s [1], [2], [3]. The prevalence of obesity (BMI ≥30 kg/m^2^) among adults 20 to 74 years of age has increased nearly threefold [2], [4], [5]. These average trends, however, fail to capture potential heterogeneous patterns in body weight over time. For example, studies have found a prominent rightward skewing of the BMI distribution over time, contributing to a larger rise in mean BMI than might be seen if the mean was the only component of the BMI distribution to change over time [2], [6], [7]. Other studies have demonstrated variability in the prevalence of overweight and obese individuals by neighborhood of residence, with greater increases in those neighborhoods with lower socioeconomic status [8], [9], [10], [11], [12].

Properly accounting for heterogeneity at both the individual and neighborhood levels using longitudinal data may determine true underlying patterns of population weight change over time with possible implications for interventions [13], [14], [15], [16]. The use of longitudinal data provides the unique opportunity to examine trajectories in BMI means and standard deviations over time and by baseline weight classification to determine what groups are at greatest risk for weight gain or have greater variability in weight gain over time.

Here, using data from the Framingham Heart Study (FHS) Offspring Cohort over 37 years, including a large number of individuals who moved great distances, we examined longitudinal trends in BMI between individuals and neighborhoods. The use of this cohort, linked together by common characteristics of their parents (or in-laws), enabled us to more confidently examine complex associations between BMI and social and geographic factors prone to endogeneity.

## Methods

### Ethics Statement

The Institutional Review Board of Harvard Medical School approved this study. The Framingham Heart Study undertook a detailed written consent process for all aspects of data collection [17].

### Sample

Our sample came from the Framingham Heart Study (FHS) Offspring Cohort, which started in 1971 and enrolled 5124 subjects who were either the children of subjects enrolled in the FHS Original Cohort or their spouses. The FHS Original Cohort included a random sample of residents of Framingham, Massachusetts, in the 1940s. Offspring Cohort subjects have been examined and surveyed up to eight times from enrollment through 2008, roughly every four years. Our final sample included all FHS Offspring Cohort subjects excluding observations with missing BMI, smoking status, alcohol intake, or census tract of residence; we also excluded subjects at any time points if they were living in a nursing home or were less than 21 years old.

**Figure 1:**
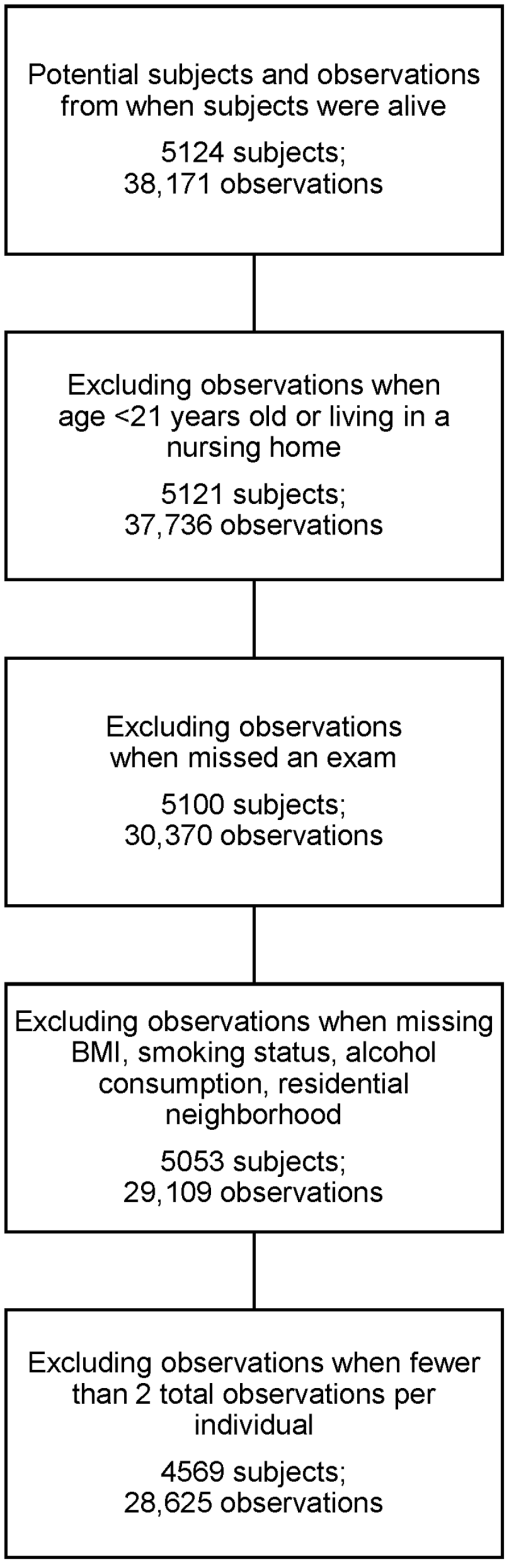
Flow Diagram for Framingham Heart Study Offspring Cohort Subjects and Observations Included in Analyses. The final sample size for this study included 4569 subjects with 28,625 observations over a nearly 40 year period.

For analyses, we intended to use three-level multi-level random effects models to account for BMI clustering by neighborhood and individual with an additional pure error variance term. However, contrary to our a priori hypothesis that we would find notable variance at the neighborhood level, we found that the neighborhood level contributed near zero variance in cross-sectional models for most of the eight waves (Table S1 in Appendix S1). Among women, the proportion of the variance contributed by the neighborhood level, calculated as the intra-class correlation coefficient (ICC) [neighborhood-level variance/(neighborhood-level variance+individual-level variance)], was less than 0.6% for five waves, and 1.5%, 1.1%, and 3.3% in Waves 5, 6 and 7, respectively. Among men, the ICC at the neighborhood level was less than 0.7% at each wave. To create the most parsimonious final models, we used only two-level multilevel models accounting for the individual-level variance and pure error variance. We included a random intercept at the individual level as well as random slopes for both time and the natural log of time. Including both fixed effects and random slopes for linear time and the natural log of time accounted for non-linearity in population-average and individual-specific BMI growth trajectories and allowed different amounts of heterogeneity between the linear and nonlinear components of individuals’ trajectories. We required subjects to have at least two observations so that each individual contributed direct information about intra-individual change in BMI across time (Figure 1). Our final sample size was 4569 subjects with 28,625 observations.

### Variables

Time-varying individual-level BMI was the outcome variable, objectively calculated using in person measured weight and standing height at each wave [18]. Individual-level covariates included the time-varying variables age, marital status, employment status, smoking status, and alcohol consumption, and also the time-invariant education (only available in Waves 2 and 3). Despite not allowing neighborhood-level variance in the final model, we did include a covariate for census tract poverty. This measure is the percent of census tract residents with family incomes below the US poverty line, and we obtained the measure from the US Census for 1970, 1980, 1990, and 2000. Its effect represents the extent to which the average neighborhood BMI covaries with the poverty level of the neighborhood after adjusting for the other characteristics of the individuals in the neighborhood. Because of changing census tract borders over time, we used data from the commercial vendor Geolytics which adjusted all census data to the 2000 tract boundaries. We assigned census data to subjects by waves according to their census tract of residence and the date of their study examination, selecting the Census closest to the examination date. Residential addresses for subjects were collected at each of the eight waves of follow-up and were subsequently geocoded using ArcGIS, Version 9.3 (Redlands, CA).

### Model Building and Analysis

Our models were two-level multilevel models accounting for between-individual and within-individual (or pure “error”) variance. When determining how best to account for individual-level variance, we explored several modeling strategies. In our data, the longitudinal trajectory in BMI appeared nonlinear with some evidence of nonlinear changes in the variance over time (Figure S1 in Appendix S1; Table S2 in Appendix S1); therefore, we chose models that included fixed effects and random slopes for both linear time and the natural log of time (see Figure S2 in Appendix S1 for model specification) to account for non-linearity. The variable time represented the wave of follow-up, with values from 1 to 8.

We first generated descriptive results using SAS statistical software, Version 9.1 (Cary, North Carolina). Using MLWin Version 2.24 (Bristol, United Kingdom) [19], we then examined the population means and the individual-level and pure error variation in BMI, controlling for individual- and neighborhood-level covariates (Methods Note S1 in Appendix S1 for details). Because of prior studies showing differential variation in BMI by gender, we ran sex-stratified models [8], [10], [12].

To determine how much of the unexplained individual-level variation in BMI was accounted for by baseline BMI, we subsequently fit separate models for Waves 2 through 8 with each model fit two ways: with Wave 1 BMI as a predictor and without. These models included all of the same covariates previously specified but had smaller sample sizes because we included outcomes only for Waves 2 through 8 (4569 subjects, 24,467 observations). We also included wave by age interactions to help differentiate temporal trends in BMI from aging trends.

Further, to determine whether BMI mean and standard deviation trajectories differed by baseline weight classification, we fit four models for each gender corresponding to the four categories of baseline BMI: underweight (BMI <18.5 kg/m^2^), normal weight (BMI 18.5 to 24.9 kg/m^2^), overweight (BMI 25 to 29.9 kg/m^2^), and obese (BMI ≥30 kg/m^2^). Our a priori hypothesis was that mean BMI increased more rapidly for overweight and obese participants [2]. In each case, a single longitudinal model was fit to BMI in Waves 2 through 8 with the same covariates, including Wave 1 BMI and wave by age interactions.

For all models, we used Markov Chain Monte Carlo (MCMC) analyses to generate multiple iterative samples from the joint posterior distribution of the parameters, from which parameter estimates could be constructed [20]. We used 10,000 iterative samples as a burn-in with 100,000 samples to generate final parameter estimates. We report posterior means and associated 95% credible intervals as point and interval estimates of the true model parameters. Significant findings are adjudicated to those predictors with estimated parameters whose 95% credible intervals excluded 0.

## Results

The mean number of observations per subject was 6.3 with a range of 2 to 8 observations (by construction, the lower limit was 2 not 1). The mean BMI increased from 24.0 kg/m^2^ at Wave 1 to 27.7 at Wave 8 for women and from 26.6 kg/m^2^ to 29.0 for men (Table 1, Figure S1 in Appendix S1).

**Table 1:** Characteristics of Sample, Framingham Heart Study Offspring Cohort, 1971 to 2008.

		Mean Across Waves	Wave 1 Mean,Wave 8 Mean	Mean Across Waves		Wave 1 Mean,Wave 8 Mean
		FemaleN = 2366*Observations = 15,016		MaleN = 2203*Observations = 13,609		
**BMI – kg/m^2^**		26.1	24.0, 27.7	27.7	26.6, 29.0	
**Age – yr**		52.4	37.3, 66.9	52.6	38.4, 67.0	
**Education – %**	**≤ High school**	44.1	45.6, 35.3	37.4	39.0, 26.5	
	**> High School**	51.8	46.9, 65.7	58.4	53.4, 73.4	
	**Missing Education**	4.1	7.5, 0	4.3	7.6, 0.1	
**Married – %**		75.4	86.2, 65.0	85.4	88.6, 84.0	
**Employed – %**		60.0	53.3, 39.4	77.3	95.6, 46.1	
**Current Smoker – %**		24.8	43.0, 8.2	24.8	45.0, 7.5	
**Alcohol Intake – %**	**0 drinks/day**	35.8	16.6, 52.0	23.8	8.8, 38.0	
	**1–2 drinks/day**	59.0	77.4, 44.9	55.2	63.9, 48.4	
	**>2 drinks/day**	5.2	6.0, 3.0	21.0	27.3, 13.6	
**Neighborhood Poverty**		5.4	6.0, 5.6	5.3	6.1, 5.4	

* The number of female subjects was 2148 in Wave 1 and 1518 in Wave 8. The number of male subjects was 2010 in Wave 1 and 1261 in Wave 8. The total number of subjects is greater than subjects in Wave 1 because some observations did not meet inclusion criteria (e.g., a subject had missing BMI in Wave 1 but available BMI in subsequent waves).

In addition to the increase in mean BMI, the *variation* in BMI increased substantially over time (Table S2 in Appendix S1, Figure S3 in Appendix S1), with higher variability at each wave for women than for men. For women, the unadjusted standard deviation increased from 4.55 kg/m^2^ in Wave 1 to 5.86 in Wave 8, and for men from 3.55 kg/m^2^ to 4.67; the values of the coefficient of variation confirm the increase in BMI variability over time and the greater variability for women. Thus, the weight diversity of the population grew across time compared to a system where the standard deviation was proportional to the mean.

The pattern of BMI distribution also changed over time, with less skewness for both women (0.04 to 0.01) and no change for men (0.02 to 0.02), indicating a more normal distribution of BMI by Wave 8 for women. Consistent with the foregoing, kurtosis, a measure of the presence of outliers, declined quite substantially over time for women (5.62 to 1.52) with a slight increase for men (1.12 to 1.76). Overall, the distribution of BMI over time maintained a similar shape for men (slightly skewed and with thicker tails than the normal distribution) but became substantially more normal for women.

The final models included all individual-level covariates as well as neighborhood poverty (Table 2). For women and men, as expected, the covariates that were positively associated with BMI were time, increasing age, increasing alcohol consumption, and being married. Mean BMI increased in a non-linear pattern for women but not for men; the natural log of time for women was significantly positively associated with BMI. Smoking and higher education (> high school vs. ≤ high school) were negatively associated with BMI for both women and men. Neighborhood poverty was not associated with BMI. For men, being employed was positively associated with BMI. Model fit did not improve with the addition of demographic variables (age, marital status, employment status, education) or with the addition of census tract poverty; however, model fit did improve with the addition of behavioral variables (alcohol consumption and smoking status) (Table S3 in Appendix S1).

**Table 2:** Parameter Estimates from Final Models, Framingham Heart Study Offspring Cohort, 1971 to 2008.

		FemaleN = 2366Obs = 15,016		MaleN = 2203Obs = 13,609	
Variable		β	95% Credible Interval	β	95% Credible Interval
**Intercept**		24.4	24.0, 24.9*	26.4	26.0, 26.8*
**Time/Wave of Observation (1 to 8)**		0.26	0.17, 0.34*	0.31	0.24, 0.38*
**Natural Log of Time**		0.61	0.38, 0.84*	0.02	−0.16, 0.21
**Age**		0.04	0.03–0.06*	0.02	0.003, 0.03*
**Education**	**≤ high school**	Ref		Ref	
	**> high school**	−0.61	−0.85, −0.36*	−0.48	−0.69, −0.27*
	**Missing education**	0.37	−0.06, 0.79	0.25	−0.10, 0.60
**Married**		0.47	0.34, 0.60*	0.26	0.14, 0.37*
**Employed**		0.12	0.03, 0.20	0.19	0.10, 0.28*
**Smoker**		−0.90	−1.0, −0.77*	−0.73	−0.84, −0.62*
**Alcohol Consumption**	**0 drinks/day**	Ref		Ref	
	**1**–**2 drinks/day**	0.19	0.10, 0.27*	0.22	0.13, 0.31*
	**>2 drinks/day**	0.29	0.10, 0.48*	0.32	0.20, 0.44*
**Neighborhood poverty** †		0.002	−0.01, 0.02	−0.006	−0.02, 0.004
**Variance Components**					
**Level**		**Standard Deviation**	**95% Credible Interval**	**Standard Deviation**	**95% Credible Interval**
**Individual Level**	**Random Intercept**	4.61	4.46, 4.75*	3.41	3.30, 3.52*
	**Random Slope for Time**	1.18	1.12, 1.24*	0.87	0.81, 0.93*
	**Random Slope for** **Natural Log of Time**	3.42	3.22, 3.62*	2.42	2.24, 2.61*
**Pure Error Variance**		1.49	1.46, 1.51*	1.25	1.23, 1.27*
**Deviance Information Criteria (DIC) for model fit**		59,538		49,167	

* 95% credible interval does not cross 0.

† Census tract information was unavailable for some tracts. Almost all of this missing data was from 1970 when some land areas were not yet assigned a census tract. For this analysis, we had census tract poverty data for 14,355 of the 15,016 observations among women and 12,989 of the 13,609 included observations among men. To ensure comparability across models, we included a dummy variable accounting for the availability of census tract poverty data along with a modified poverty variable (missing poverty data set to 0 rather than missing) in the final model. This did not change results for census tract poverty but did allow us to include all observations in the analyses that included this variable.

**Figure 2:**
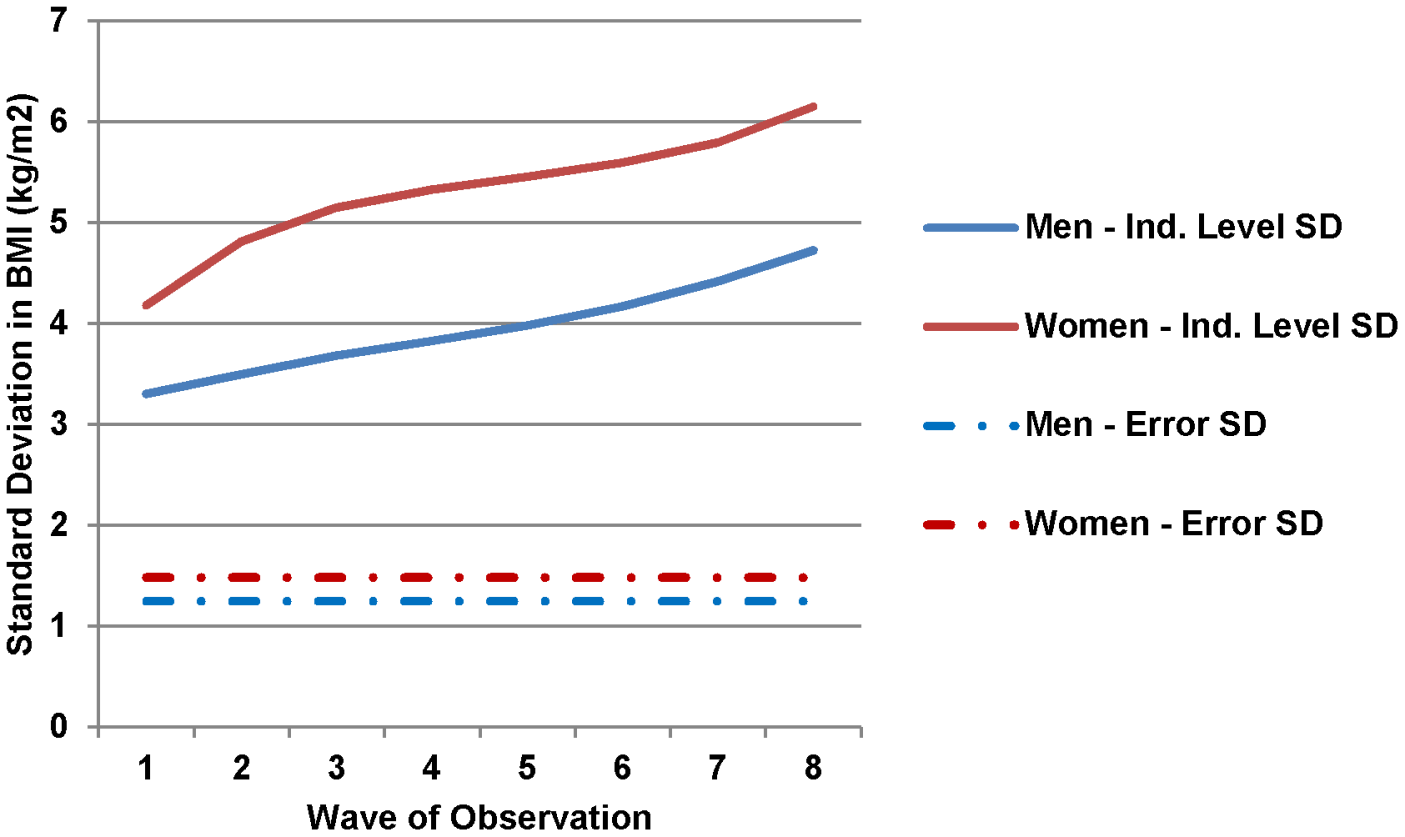
Adjusted Standard Deviation in Body Mass Index, Framingham Heart Study Offspring Cohort, 1971–2008. In the fully adjusted models, the total unexplained variation in BMI attributed to individuals across time (individual-level standard deviation) steadily increased from 1971 to 2008 for both women and men. The error standard deviation represents the idiosyncratic pure error variance. We accounted for non-linear increases in between-individual BMI standard deviation by including a random intercept at the individual level and random slopes for time and the natural log of time.

As we found for the unadjusted BMI variance, the individual-level random slopes for time and the natural log of time in fully-adjusted models revealed increasing heterogeneity in BMI across time for women and men (Table 2, Figure 2). The adjusted standard deviation in BMI increased more than the unadjusted, from 4.18 kg/m^2^ at Wave 1 to 6.15 at Wave 8 for women and from 3.31 kg/m^2^ to 4.73 for men (Figure 2). Thus, similar to unadjusted BMI, we found a greater increase in the BMI variance than the BMI mean after controlling for covariates and clustering.

To determine how much of the between-individual variation in BMI was accounted for by baseline BMI at Wave 1, we ran two sets of models restricted to observations from Waves 2 to 8, with baseline BMI and without, including all of the same covariates as for the prior models. In models without baseline BMI, the standard deviation in BMI at Wave 2 was 4.63 kg/m^2^ for women and 3.46 for men (variance 21.5 kg/m^2^ and 12.0). The addition of baseline BMI decreased standard deviations at Wave 2 to 1.95 kg/m^2^ for women and 1.42 for men (variance 3.80 kg/m^2^ and 2.01). The baseline BMI, thus, accounted for 82% and 83% of the between-individual variance in BMI, respectively, for women and men (data not shown in tables).

To assess the impact of baseline weight on the trajectories of BMI mean and variance, we then fit four models for each sex, stratified by baseline BMI classification – underweight, normal weight, overweight, obese - including BMI in Waves 2 to 8 as the outcome. These models also included wave by age interactions and Wave 1 BMI as predictors (Table S4 in Appendix S1, Table S5 in Appendix S1, Table S6 in Appendix S1, Table S7 in Appendix S1). In these models, being married was associated with higher BMI for normal weight and overweight women and men as well as obese women. Smoking was negatively associated with BMI in nearly all models, showing its strong negative effect on weight gain over time. Alcohol consumption was associated with higher weight only among overweight women and men and normal weight men. Higher baseline BMI was associated with higher subsequent BMI except among underweight women and men. The interaction effects between age and wave were negative and significant in nearly all models, suggesting that age became an increasingly protective factor against weight gain over time. This trend likely represents the transition from being young adults (when metabolic rate and exercise levels may decline with age) to elderly (when reduced muscle mass and frailty may overpower reductions in metabolic rate).

**Figure 3:**
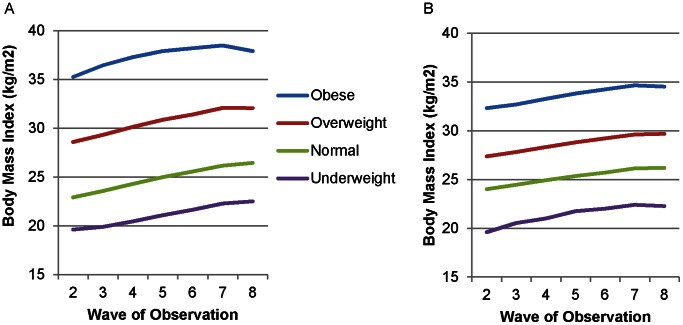
Body Mass Index Trajectories by Baseline Weight Classification, Framingham Heart Study Offspring Cohort, 1979–2008. Using results from the fully-adjusted models, we plotted the BMI trajectory for women (A) and men (B) based on their weight classification at baseline (during Wave 1, 1971–1975), controlling for covariates including baseline BMI. Weight classifications were underweight (BMI <18.5 kg/m^2^), normal weight (18.5 to 24.9), overweight (25 to 29.9), and obese (≥30). Lines represent trajectories for the typical male or female (mean age at each wave, married, employed,>high school education, non-smoker, consuming 1–2 alcoholic drinks daily, living in a census tract at mean poverty level, with mean baseline BMI for that weight classification).

**Figure 4:**
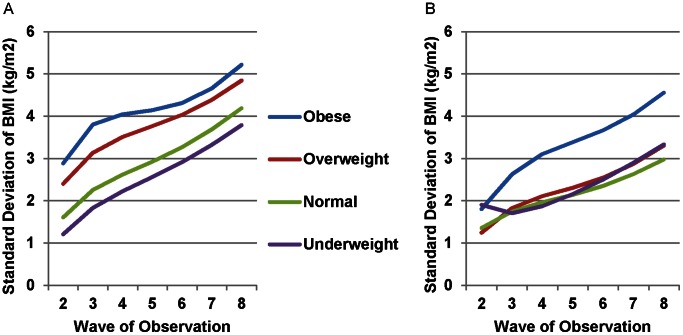
Individual-Level Standard Deviation in Body Mass Index by Baseline Weight Classification, Framingham Heart Study Offspring Cohort, 1979–2008. In the fully adjusted models, the individual-level standard deviation of BMI steadily increased from 1971 to 2008 for both women (A) and men (B) in all baseline weight classifications. Standard deviation increases were similar across most weight classifications with larger standard deviations for both obese women and men, and larger increases across time for obese men. We accounted for non-linear increases in between-individual BMI standard deviation by including a random intercept at the individual level and random slopes for time and the natural log of time.

In these models, mean BMI among men had parallel increases across baseline weight classifications with a plateau in BMI evident by Wave 8 (Figure 3B). Women had similar patterns except for obese women, who demonstrated somewhat more rapid weight gain in initial waves, followed by a partial reversal by Wave 8 (Figure 3A). Among women, standard deviation was proportion to baseline weight, with highest standard deviations for obese women over time and lowest for underweight women (Figure 4A). For men, underweight, normal weight, and overweight subjects at baseline had very similar standard deviations over time (Figure 4B). Obese men had substantially higher standard deviations with continued divergence of these values from other weight classes over time.

## Discussion

Using data from the Framingham Heart Study Offspring Cohort over a nearly 40 year period, we show that factors intrinsic to individuals accounted for the overwhelming proportion of the variation in BMI over time. We also found increasing population means and variation for BMI over time. For both men and women, baseline BMI accounted for most of the unexplained individual-level variation in BMI, demonstrating that BMI reached by the late 30 s (mean age at Wave 1 was 38 years for men, 37 for women), determined BMI until their late 60 s (mean age at Wave 8 was 67 years for both men and women). The rapidity of weight gain was similar across all baseline weight classifications except for women who were obese at baseline. Obese women gained weight somewhat more rapidly than women with lower baseline BMIs until they were in their early 50 s with an abatement of this trend thereafter. BMI variation increased over time for participants in all baseline weight categories. Variation was greatest for obese female and male subjects, demonstrating a more heterogeneous population across time.

The parallel increases in weight gain across baseline weight classifications calls for a relatively uniform population-targeted strategy to decrease risk for weight gain. Further, because weight trajectories appear to be set by the late 30 s, strategies focused on children and young adults might be most effective [21]. The more rapid increases in BMI through middle age among obese women call for somewhat varied strategies to address risk for weight gain by age. Obese women may benefit from more aggressive interventions to counter risk for weight gain during middle age, with less need for interventions in the mid-to-late 60 s due to a typical regression of weight gain by that point. Men have similar BMI increases across time irrespective of baseline BMI; however, the more rapid increase in variance among obese men also calls for somewhat more targeted approaches for this group.

These results, showing increasing variation in BMI but a more uniform distribution over time, contrasts somewhat with recent data from Flegal, et al. [2]. That study used data from the National Health and Nutrition Examination Survey (NHANES), a repeated cross-sectional survey of a representative sample of US adults, and found an increase in BMI mean and variation as well as a rightward skewing of the distribution of BMI over time for both women and men. Using our large longitudinal database, and accounting for both aging and secular trends, we find an increase in BMI mean and variation, with a more normal distribution of BMI emerging across time, especially for women.

Finally, our analyses shed light on the possible role of neighborhood of residence in the growth of obesity over the past four decades. In contrast to prior longitudinal studies, in our study, neighborhood of residence accounted for a very small proportion of BMI variance, and neighborhood poverty was unrelated to BMI [11], [22]. Because of the very small variance contributed by the neighborhood level in cross sectional models in most waves, we did not include neighborhood as a level in final models. We did find that census tracts accounted for 1% or more of the total variation in BMI for women during three waves; however, in the other five waves, neighborhoods accounted for less than 0.6% of the total BMI variation. Finding these differences across time highlights the importance of having longitudinal data for a cohort over a long period of time. Our study may differ from prior studies because of the characteristics of our sample, which included racially homogeneous subjects mostly living in smaller towns where public transportation is limited, typically requiring use of cars for transportation.

Our study has limitations. First, we could not measure characteristics of neighborhoods where subjects work, a possible source of unmeasured confounding between BMI and neighborhood characteristics. Second, we could more effectively determine the age at which BMI trajectories are established if we had measurements prior to the 1970s. Third, our sample lacks racial diversity, an unavoidable limitation of research with the FHS Offspring Cohort. However, this limitation in generalizability also could strengthen the plausibility of our findings. All subjects had some similar characteristics because they are the offspring (or an offspring’s spouse) of the FHS Original Cohort, a random sampling of Framingham, Massachusetts, in the 1940s. One could argue that with fewer differences between individuals on observables, such as race, that it is reasonable to assume there are also fewer differences on unobservables and thus less impact from unmeasured confounding. Further, subjects were socioeconomically quite diverse. For example, in Wave 8, the mean census tract poverty for male subjects was 5.4% (SD 4.3%, Range 0.3% –31.0%). Fourth, we had a large number of census tracts in our sample, frequently with a small number of observations per tract. Our sample included participants from 2095 different census tracts over time, with a mean of 13.7 observations per tract (SD 79.8, range 1 to 1638). Multilevel models, by design, shrink the variance estimates toward the null for higher level units (tracts) with few observations and, therefore, may underestimate the ICC at the tract level in the cross-sectional models that we ran. Yet, shrunken residuals have the benefit of helping to avoid over-interpretation of random variation in the data as true neighborhood-level variation.

In sum, over nearly 40 years, BMI mean and variation increased in parallel across most baseline weight classifications in our sample. Individual-level characteristics, especially baseline BMI, were the primary factors in rising BMI. These findings have important implications not only for understanding the sources of the obesity epidemic in the United States but also for the targeting of interventions to address the epidemic.

## Supporting Information

Appendix S1 Table S1, Cross-sectional Variance at the Neighborhood and Individual Levels, by Wave. Table S2, Unadjusted Skewness, Kurtosis and Coefficients of Variation by Wave. Table S3, Deviance Information Criteria (DIC) for Models. Table S4, Parameter Estimates from Models for Participants Who Were Underweight (BMI <18.5 kg/m^2^) at Baseline (1971 to 75) Followed from 1979 to 2008 to Examine BMI Trajectories, Framingham Heart Study Offspring Cohort. Table S5, Parameter Estimates from Models for Participants Who Were Normal Weight (BMI 18.5 to 24.9 kg/m^2^) at Baseline (1971 to 75) Followed from 1979 to 2008 to Examine BMI Trajectories, Framingham Heart Study Offspring Cohort. Table S6, Parameter Estimates from Models for Participants Who Were Overweight (BMI 25.0 to 29.9 kg/m^2^) at Baseline (1971 to 75) Followed from 1979 to 2008 to Examine BMI Trajectories, Framingham Heart Study Offspring Cohort. Table S7, Parameter Estimates from Models for Participants Who Were Obese (BMI ≥30 kg/m^2^) at Baseline (1971 to 75) Followed from 1979 to 2008 to Examine BMI Trajectories, Framingham Heart Study Offspring Cohort. Figure S1, Mean Body Mass Index for Women and Men, Framingham Heart Study Offspring Study, 1971 to 2008. Mean unadjusted BMI increased for both women and men over the course of follow-up with a more steep trajectory for women than men. Figure S2, Model for Primary Analyses Examining Body Mass Index, Framingham Heart Study Offspring Cohort Study, 1971 to 2008. We generated this screen shot from MLWin to display the model we ran for our primary analyses, demonstrating both the fixed and random effects included. This example is for our full sample of women, but the models were equivalent for men. In these models, we include a fixed and random effect for linear time (linear time from 1 to 8, based on wave of observation (time)), the natural log of time (lntime), age (a linear variable centered on its mean (age-gm)), marital status (binary: unmarried as reference, married (married_1)), education (categorical: ≤high school as reference, >high school (educat_1), missing education (educat_2)), employment status (binary: unemployed as reference, employed (employed_1)), smoking status (binary: non-smoker as reference, smoker (smokes_1)), alcohol consumption (categorical: 0 as reference, 1–2 daily (alcgrp_1), >2 daily (alcgrp_2)), census tract poverty (linear variable centered on its mean (newpov-gm)), and whether census tract poverty was available (binary: not available as reference, available (povavail_1)). We included this last variable to allow us to have equal subjects in models with census tract poverty in the model and those without it. Models that we stratified by baseline BMI classification were similar, but the outcome in these models was BMI from Waves 2 through 8 (rather than 1 through 8) and included baseline BMI and age by time interactions as additional covariates. Figure S3, Histogram of Unadjusted BMI Distribution for Subjects in Wave 1 (1971 to 1975, Diagonal Stripes) and Wave 8 (2005 to 2008, Open Bars). These histograms represent the distribution of BMI values for Wave 1 versus Wave 8, demonstrating an increase in BMI mean and variance for both women (A) and men (B). Methods Note S1.
(DOC)
