# Supplementary material for: Population Trends and Variation in Body Mass Index from 1971 to 2008 in the Framingham Heart Study Offspring Cohort
Source: PLoS One. 2013 May 10;8(5):e63217. doi: 10.1371/journal.pone.0063217 (PMC3651246; doi:10.1371/journal.pone.0063217)
Supplement: Appendix S1 — Table S1, Cross-sectional Variance at the Neighborhood and Individual Levels, by Wave. Table S2, Unadjusted Skewness, Kurtosis and Coefficients of Variation by Wave. Table S3, Deviance Information Criteria (DIC) for Models. Table S4, Parameter Estimates from Models for Participants Who Were Underweight (BMI <18.5 kg/m2) at Baseline (1971 to 75) Followed from 1979 to 2008 to Examine BMI Trajectories, Framingham Heart Study Offspring Cohort. Table S5, Parameter Estimates from Models for Participants Who Were Normal Weight (BMI 18.5 to 24.9 kg/m2) at Baseline (1971 to 75) Followed from 1979 to 2008 to Examine BMI Trajectories, Framingham Heart Study Offspring Cohort. Table S6, Parameter Estimates from Models for Participants Who Were Overweight (BMI 25.0 to 29.9 kg/m2) at Baseline (1971 to 75) Followed from 1979 to 2008 to Examine BMI Trajectories, Framingham Heart Study Offspring Cohort. Table S7, Parameter Estimates from Models for Participants Who Were Obese (BMI ≥30 kg/m2) at Baseline (1971 to 75) Followed from 1979 to 2008 to Examine BMI Trajectories, Framingham Heart Study Offspring Cohort. Figure S1, Mean Body Mass Index for Women and Men, Framingham Heart Study Offspring Study, 1971 to 2008. Mean unadjusted BMI increased for both women and men over the course of follow-up with a more steep trajectory for women than men. Figure S2, Model for Primary Analyses Examining Body Mass Index, Framingham Heart Study Offspring Cohort Study, 1971 to 2008. We generated this screen shot from MLWin to display the model we ran for our primary analyses, demonstrating both the fixed and random effects included. This example is for our full sample of women, but the models were equivalent for men. In these models, we include a fixed and random effect for linear time (linear time from 1 to 8, based on wave of observation (time)), the natural log of time (lntime), age (a linear variable centered on its mean (age-gm)), marital status (binary: unmarried as reference, married (married_ [file pone.0063217.s001.doc]

**Table S1 in Appendix S1: Cross-sectional Variance at the Neighborhood and Individual Levels, by Wave**

**Table S2 in Appendix S1: Unadjusted Skewness, Kurtosis and Coefficients of Variation by Wave**

**Table S3 in Appendix S1: Deviance Information Criteria (DIC) for Models**

**Table S4 in Appendix S1: Parameter Estimates from Models for Participants Who Were Underweight (BMI <18.5 kg/m^2^) at Baseline (1971 to 75) Followed from 1979 to 2008 to Examine BMI Trajectories, Framingham Heart Study Offspring Cohort**

**Table S5 in Appendix S1: Parameter Estimates from Models for Participants Who Were Normal Weight (BMI 18.5 to 24.9 kg/m^2^) at Baseline (1971 to 75) Followed from 1979 to 2008 to Examine BMI Trajectories, Framingham Heart Study Offspring Cohort**

**Table S6 in Appendix S1: Parameter Estimates from Models for Participants Who Were Overweight (BMI 25.0 to 29.9 kg/m^2^) at Baseline (1971 to 75) Followed from 1979 to 2008 to Examine BMI Trajectories, Framingham Heart Study Offspring Cohort**

**Table S7 in Appendix S1: Parameter Estimates from Models for Participants Who Were Obese (BMI ≥ 30 kg/m^2^) at Baseline (1971 to 75) Followed from 1979 to 2008 to Examine BMI Trajectories, Framingham Heart Study Offspring Cohort**

**Figure S1 in Appendix S1: Mean Body Mass Index for Women and Men, Framingham Heart Study Offspring Study, 1971 to 2008**

**Figure S2 in Appendix S1: Model for Primary Analyses Examining Body Mass Index, Framingham Heart Study Offspring Cohort Study, 1971 to 2008**

**Figure S3 in Appendix S1: Histogram of Unadjusted BMI Distribution for Subjects in Wave 1 (1971 to 1975, Diagonal Stripes) and Wave 8 (2005 to 2008, Open Bars)**

**Methods Note S1 in Appendix S1**

**Table S1 in Appendix S1**: Cross-sectional Variance at the Neighborhood and Individual Levels, by Wave

|  | **Female** | | | | **Male** | | | |
| --- | --- | --- | --- | --- | --- | --- | --- | --- |
|  | **Individual Level** | **95% Credible Interval** | **Neighborhood Level** | **95% Credible Interval** | **Individual Level Variance** | **95% Credible Interval** | **Neighborhood Level Variance** | **95% Credible Interval** |
| **Wave 1** | 20.7 | 19.4, 22.0 | 0.06 | 0.001, 0.34 | 12.6 | 11.9 | 0.03 | 0.001, 0.18 |
| **Wave 2** | 23.5 | 22, 25.0 | 0.12 | 0.001, 0.54 | 13.3 | 12.5 | 0.04 | 0.001, 0.24 |
| **Wave 3** | 27.6 | 25.9, 29.4 | 0.15 | 0.001, 0.68 | 14.2 | 13.3 | 0.05 | 0.001, 0.27 |
| **Wave 4** | 30.3 | 28.4, 32.3 | 0.17 | 0.001, 0.75 | 15.1 | 14.1 | 0.1 | 0.001, 0.44 |
| **Wave 5** | 30.3 | 28.4, 32.3 | 0.45 | 0.006, 1.25 | 17.2 | 16 | 0.12 | 0.001, 0.49 |
| **Wave 6** | 31.8 | 29.7, 34.0 | 0.37 | 0.001, 1.33 | 19.3 | 18 | 0.07 | 0.001, 0.39 |
| **Wave 7** | 33.3 | 31, 35.7 | 1.13 | 0.11, 2.57 | 20.6 | 19.2 | 0.05 | 0.001, 0.3 |
| **Wave 8** | 34.3 | 31.9, 36.8 | 0.14 | 0.001, 0.79 | 21.8 | 20.1 | 0.09 | 0.001, 0.54 |

**Table S2 in Appendix S1: Unadjusted** Skewness, Kurtosis and Coefficients of Variation by Wave

| **Female** | | | | | **Male** | | | |
| --- | --- | --- | --- | --- | --- | --- | --- | --- |
|  | **Standard Deviation** | **Skewness** | **Kurtosis** | **Coefficient of Variation** | **Standard Deviation** | **Skewness** | **Kurtosis** | **Coefficient of Variation** |
| **Wave 1** | 4.55 | 0.04 | 5.62 | 0.19 | 3.55 | 0.02 | 1.12 | 0.13 |
| **Wave 2** | 4.85 | 0.03 | 3.04 | 0.20 | 3.66 | 0.03 | 1.95 | 0.14 |
| **Wave 3** | 5.26 | 0.02 | 3.81 | 0.21 | 3.77 | 0.03 | 1.50 | 0.14 |
| **Wave 4** | 5.51 | 0.02 | 3.72 | 0.21 | 3.89 | 0.03 | 1.52 | 0.14 |
| **Wave 5** | 5.54 | 0.01 | 1.79 | 0.21 | 4.16 | 0.03 | 1.90 | 0.15 |
| **Wave 6** | 5.67 | 0.01 | 2.10 | 0.21 | 4.40 | 0.02 | 1.81 | 0.15 |
| **Wave 7** | 5.86 | 0.01 | 2.83 | 0.21 | 4.54 | 0.02 | 2.37 | 0.16 |
| **Wave 8** | 5.86 | 0.01 | 1.52 | 0.21 | 4.67 | 0.02 | 1.76 | 0.16 |

**Table S3 in Appendix S1: Deviance Information Criteria (DIC)* for M**odels

| **Models** | Female  N= 2,366  Obs5,016 | Male  N= 2,203  Obs=13,609 |
| --- | --- | --- |
| Time and natural log of time as linear variables | 59,743 | 49,329 |
| + age, sex, marital status, education, employment status | 59,758 | 49,328 |
| +alcohol consumption, smoking status | 59,540 | 49,179 |
| + neighborhood poverty^†^ | 59,538 | 49,167 |

* Model fit and complexity were evaluated using the Deviance Information Criterion (DIC), which is similar to Akaike’s Information Criterion (AIC).

† Each successive model presented is additive such that the final model (+neighborhood poverty) includes all of the variables. Census tract information was unavailable for some tracts. Almost all of this missing data was from 1970 when some land areas were not yet assigned a census tract. For this analysis, we had census tract poverty data for 12,989 of the 13,609 included observations among men and 14,355 of the 15,016 observations among women. To ensure comparability across models especially with regards to the DIC, we included a dummy variable accounting for the availability of census tract poverty data along with a modified poverty variable (missing poverty data set to 0 rather than missing) in the final model. This did not change results for census tract poverty but did allow us to include all observations in the analyses that included this variable.

**Table S4 in Appendix S1: Parameter Estimates from Models for Participants Who Were Underweight (BMI <18.5 kg/m2) at Baseline (1971 to 75) Followed from 1979 to 2008 to Examine BMI Trajectories, Framingham Heart Study Offspring Cohort**†

| **Variable** | | | **β** | **95% Credible**  **Interval*** | **β** | **95% Credible**  **Interval*** |
| --- | --- | --- | --- | --- | --- | --- |
|  | | | Female  N= 80  Obs= 425 | | Male  N= 27  Obs= 147 | |
| Intercept | | | 17.3 | 16.2, 18.4* | 16.8 | 14.4, 19.3* |
| Time | | | 1.12 | 0.69, 1.56* | 1.00 | 0.37, 1.60* |
| Natural Log of Time | | | -1.26 | -2.45, -0.13* | -0.16 | -1.82, 1.54 |
| Baseline BMI (centered on mean) | | | -0.05 | -0.53, 0.42 | 0.67 | -0.06, 1.34 |
| Age (centered on mean) | | | -0.004 | -0.05, 0.04 | -0.11 | -0.19, -0.02* |
| Education | ≤ high school | | Ref | | Ref | |
|  | > high school | | 0.45 | -0.21, 1.13 | 1.10 | -0.02, 2.28 |
|  | Missing education | | 1.60 | 0.16, 2.96* | 0.75 | -0.94, 2.47 |
| Married | | | 0.31 | -0.13, 0.74 | 0.54 | -0.24, 1.31 |
| Employed | | | 0.09 | -0.32, 0.49 | -0.55 | -1.40, 0.29 |
| Smoker | | | -0.35 | -0.84, 0.13 | -1.14 | -1.85, -0.41* |
| Alcohol Consumption | 0 drinks/day | | Ref | | Ref | |
|  | 1-2 drinks/day | | 0.15 | -0.18, 0.50 | -0.16 | -1.00, 0.71 |
|  | >2 drinks/day | | -0.03 | -0.72, 0.65 | 0.05 | -0.91, 1.02 |
| Neighborhood poverty (centered on mean) | | | 0.002 | -0.05, 0.05 | 0.06 | -0.01, 0.13 |
| Age x Time | | | -0.01 | -0.02, -0.002* | -0.01 | -0.02, 0.01 |
| **Variance Components** | | | | | | |
| **Level** | | | **Standard Deviation** | **95% Credible Interval** | **Standard Deviation** | **95% Credible Interval** |
| Individual Level | | Intercept | 1.58 | 1.13, 2.05* | 1.70 | 1.09, 2.42* |
|  | | Random Slope for Time | 0.87 | 0.62, 1.14* | 0.66 | 0.39, 1.01* |
|  | | Random Slope for Natural Log of Time | 1.99 | 1.30, 2.78* | 1.65 | 0.87, 2.66* |
| Observation Level | | | 1.05 | 0.96, 1.16* | 0.90 | 0.77, 1.05* |

* 95% credible interval does not cross 0

† We stratified the sample by the four baseline BMI groups. We excluded the Wave 1 observations from these analyses because we controlled for baseline BMI and because the residual baseline variability in BMI does not represent a meaningful steady state.

**Table S5 in Appendix S1: Parameter Estimates from Models for Participants Who Were Normal Weight (BMI 18.5 to 24.9 kg/m^2^) at Baseline (1971 to 75) Followed from 1979 to 2008 to Examine BMI Trajectories, Framingham Heart Study Offspring Cohort**^†^

| **Variable** | | | **β** | **95% Credible**  **Interval*** | **β** | **95% Credible**  **Interval*** |
| --- | --- | --- | --- | --- | --- | --- |
|  | | | Female  N= 1620  Obs= 9000 | | Male  N= 772  Obs= 4092 | |
| Intercept | | | 19.2 | 13.7, 24.8* | 24.0 | 22.1, 25.9* |
| Time | | | 0.94 | 0.82, 1.06* | 0.67 | 0.54, 0.79* |
| Natural Log of Time | | | -0.57 | -0.84, -0.30* | -0.23 | -0.55, 0.08 |
| Baseline BMI (centered on mean) | | | 0.97 | 0.91, 1.03* | 0.79 | 0.71, 0.87* |
| Age (centered on mean) | | | 0.02 | 0.006, 0.03* | -0.02 | -0.04, -0.01* |
| Education | ≤ high school | | Ref | | Ref | |
|  | > high school | | -0.02 | -0.19, 0.16 | -0.18 | -0.40, 0.04 |
|  | Missing education | | 0.23 | -0.12, 0.59 | 0.41 | 0.007, 0.80* |
| Married | | | 0.24 | 0.11, 0.37* | 0.19 | 0.03, 0.34* |
| Employed | | | -0.02 | -0.10, 0.07 | 0.04 | -0.10, 0.17 |
| Smoker | | | -0.68 | -0.80, -0.55* | -0.72 | -0.87, -0.56* |
| Alcohol Consumption | 0 drinks/day | | Ref | | Ref | |
|  | 1-2 drinks/day | | 0.04 | -0.05, 0.12 | 0.21 | 0.09, 0.33* |
|  | >2 drinks/day | | 0.10 | -0.09, 0.28 | 0.27 | 0.11, 0.43* |
| Neighborhood poverty (centered on mean) | | | 0.01 | -0.002, 0.02 | -0.01 | -0.02, 0.006 |
| Age x Time | | | -0.02 | -0.02, -0.01* | -0.01 | -0.01, -0.007* |
| **Variance Components** | | | | | | |
| **Level** | | | **Standard Deviation** | **95% Credible Interval** | **Standard Deviation** | **95% Credible Interval** |
| Individual Level | | Intercept | 2.04 | 1.94, 2.14* | 1.67 | 1.55, 1.79* |
|  | | Random Slope for Time | 1.00 | 0.93, 1.08* | 0.75 | 0.66, 0.84* |
|  | | Random Slope for Natural Log of Time | 2.14 | 1.89, 2.37* | 1.63 | 1.34, 1.91* |
| Observation Level | | | 1.20 | 1.18, 1.23* | 0.94 | 0.92, 0.97* |

* 95% credible interval does not cross 0

† We stratified the sample by the four baseline BMI groups. We excluded the Wave 1 observations from these analyses because we controlled for baseline BMI and because the residual baseline variability in BMI does not represent a meaningful steady state.

**Table S6 in Appendix S1: Parameter Estimates from Models for Participants Who Were Overweight (BMI 25.0 to 29.9 kg/m^2^) at Baseline (1971 to 75) Followed from 1979 to 2008 to Examine BMI Trajectories, Framingham Heart Study Offspring Cohort**^†^

| **Variable** | | | **β** | **95% Credible**  **Interval*** | **β** | **95% Credible**  **Interval*** |
| --- | --- | --- | --- | --- | --- | --- |
|  | | | Female  N= 446  Obs= 2387 | | Male  N= 1076  Obs= 5697 | |
| Intercept | | | 26.3 | 25.7, 26.9* | 25.7 | 22.5, 28.9* |
| Time | | | 1.19 | 0.88, 1.50* | 0.77 | 0.62, 0.91* |
| Natural Log of Time | | | -0.79 | -1.50, -0.08* | -0.54 | -0.87, -0.22* |
| Baseline BMI (centered on mean) | | | 1.09 | 0.89, 1.29* | 0.94 | 0.86, 1.01* |
| Age (centered on mean) | | | 0.01 | -0.02, 0.04 | 0.01 | -0.004, 0.02 |
| Education | ≤ high school | | Ref | | Ref | |
|  | > high school | | 0.01 | -0.49, 0.53 | -0.12 | -0.32, 0.07 |
|  | Missing education | | 0.004 | -1.05, 1.03 | 0.57 | 0.12, 1.04* |
| Married | | | 0.55 | 0.19, 0.92* | 0.20 | 0.02, 0.39* |
| Employed | | | 0.20 | -0.05, 0.45 | 0.03 | -0.10, 0.17 |
| Smoker | | | -1.17 | -1.53, -0.80* | -0.51 | -0.66, -0.35* |
| Alcohol Consumption | 0 drinks/day | | Ref | | Ref | |
|  | 1-2 drinks/day | | 0.21 | -0.02, 0.44 | 0.10 | -0.03, 0.23 |
|  | >2 drinks/day | | 0.56 | 0.03, 1.12* | 0.18 | 0.01, 0.35* |
| Neighborhood poverty (centered on mean) | | | -0.01 | -0.05, 0.03 | 0.001 | -0.02, 0.02 |
| Age x Time | | | -0.03 | -0.03, -0.02* | -0.02 | -0.02, -0.01* |
| **Variance Components** | | | | | | |
| **Level** | | | **Standard Deviation** | **95% Credible Interval** | **Standard Deviation** | **95% Credible Interval** |
| Individual Level | | Intercept | 2.92 | 2.65, 3.20* | 1.73 | 1.61, 1.84* |
|  | | Random Slope for Time | 1.25 | 1.05, 1.46* | 1.01 | 0.91, 1.10* |
|  | | Random Slope for Natural Log of Time | 3.03 | 2.40, 3.67* | 2.35 | 2.06, 2.64* |
| Observation Level | | | 1.71 | 1.64, 1.78* | 1.21 | 1.18, 1.24* |

* 95% credible interval does not cross 0

† We stratified the sample by the four baseline BMI groups. We excluded the Wave 1 observations from these analyses because we controlled for baseline BMI and because the residual baseline variability in BMI does not represent a meaningful steady state.

**Table S7 in Appendix S1: Parameter Estimates from Models for Participants Who Were Obese (BMI ≥ 30 kg/m^2^) at Baseline (1971 to 75) Followed from 1979 to 2008 to Examine BMI Trajectories, Framingham Heart Study Offspring Cohort^†^**

| **Variable** | | | **β** | **95% Credible**  **Interval*** | **β** | **95% Credible**  **Interval*** |
| --- | --- | --- | --- | --- | --- | --- |
|  | | | Female  N= 220  Obs= 1056 | | Male  N= 328  Obs= 1663 | |
| Intercept | | | 33.8 | 32.6, 35.0* | 31.2 | 30.4, 32.0* |
| Time | | | 0.87 | 0.23, 1.50* | 1.10 | 0.72, 1.47* |
| Natural Log of Time | | | 0.44 | -1.07, 1.94 | -1.35 | -2.26, -0.43* |
| Baseline BMI (centered on mean) | | | 0.79 | 0.68, 0.91* | 0.94 | 0.83, 1.05* |
| Age (centered on mean) | | | -0.01 | -0.08, 0.06 | 0.04 | 0.005, 0.07* |
| Education | ≤ high school | | Ref | | Ref | |
|  | > high school | | -1.05 | -2.04, -0.03* | 0.34 | -0.15, 0.84 |
|  | Missing education | | -0.06 | -1.74, 1.61 | 0.22 | -0.95, 1.39 |
| Married | | | 0.82 | 0.08, 1.58* | -0.49 | -1.01, 0.04 |
| Employed | | | -0.12 | -0.69, 0.44 | -0.04 | -0.38, 0.31 |
| Smoker | | | -1.23 | -2.02, -0.46* | -0.42 | -0.83, -0.005* |
| Alcohol Consumption | 0 drinks/day | | Ref | | Ref | |
|  | 1-2 drinks/day | | 0.49 | -0.03, 1.00 | 0.30 | -0.04, 0.63 |
|  | >2 drinks/day | | 0.53 | -0.78, 1.81 | 0.33 | -0.11, 0.76 |
| Neighborhood poverty (centered on mean) | | | -0.04 | -0.11, 0.04 | 0.01 | -0.03, 0.05 |
| Age x Time | | | -0.04 | -0.05, -0.02* | -0.03 | -0.04, -0.02* |
| **Variance Components** | | | | | | |
| **Level** | | | **Standard Deviation** | **95% Credible Interval** | **Standard Deviation** | **95% Credible Interval** |
| Individual Level | | Intercept | 4.04 | 3.47, 4.64* | 2.23 | 1.93, 2.54* |
|  | | Random Slope for Time | 1.61 | 1.25, 1.97* | 1.46 | 1.21, 1.71* |
|  | | Random Slope for Natural Log of Time | 3.34 | 2.46, 4.31* | 3.91 | 3.16, 4.66* |
| Observation Level | | | 2.66 | 2.52, 2.81* | 1.77 | 1.69, 1.86* |

* 95% credible interval does not cross 0

† We stratified the sample by the four baseline BMI groups. We excluded the Wave 1 observations from these analyses because we controlled for baseline BMI and because the residual baseline variability in BMI does not represent a meaningful steady state.

**Figure S1 in Appendix S1: Unadjusted Mean Body Mass Index for Women and Men, Framingham Heart Study Offspring Study, 1971 to 2008**

**
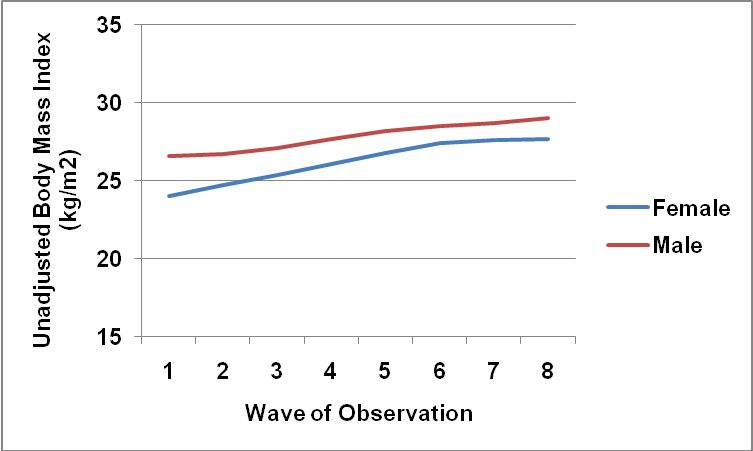
**

**Figure S2 in Appendix S1: Model for Primary Analyses Examining Body Mass Index, Framingham Heart Study Offspring Cohort Study, 1971 to 2008**

**
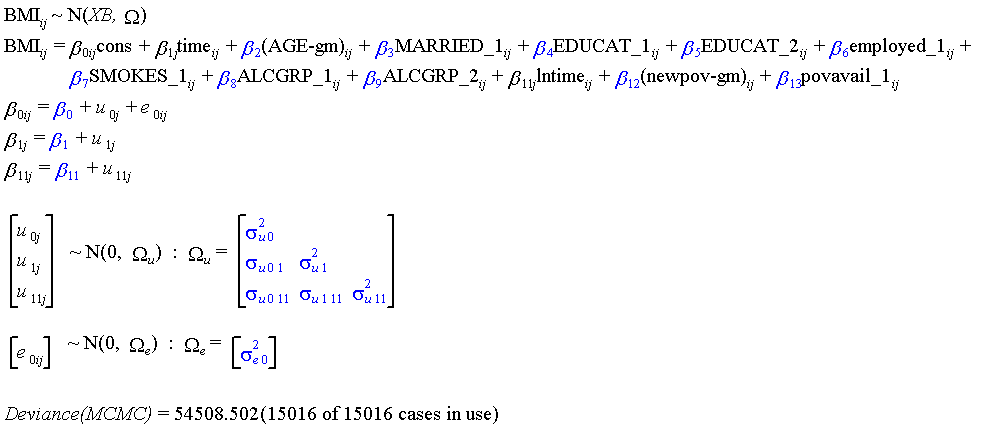
**

**Figure S3 in Appendix S1: Histogram of Unadjusted BMI Distribution for Women (A) and Men (B) in Wave 1 (1971 to 1975, Diagonal Stripes) and Wave 8 (2005 to 2008, Open Bars)**

**A**

**
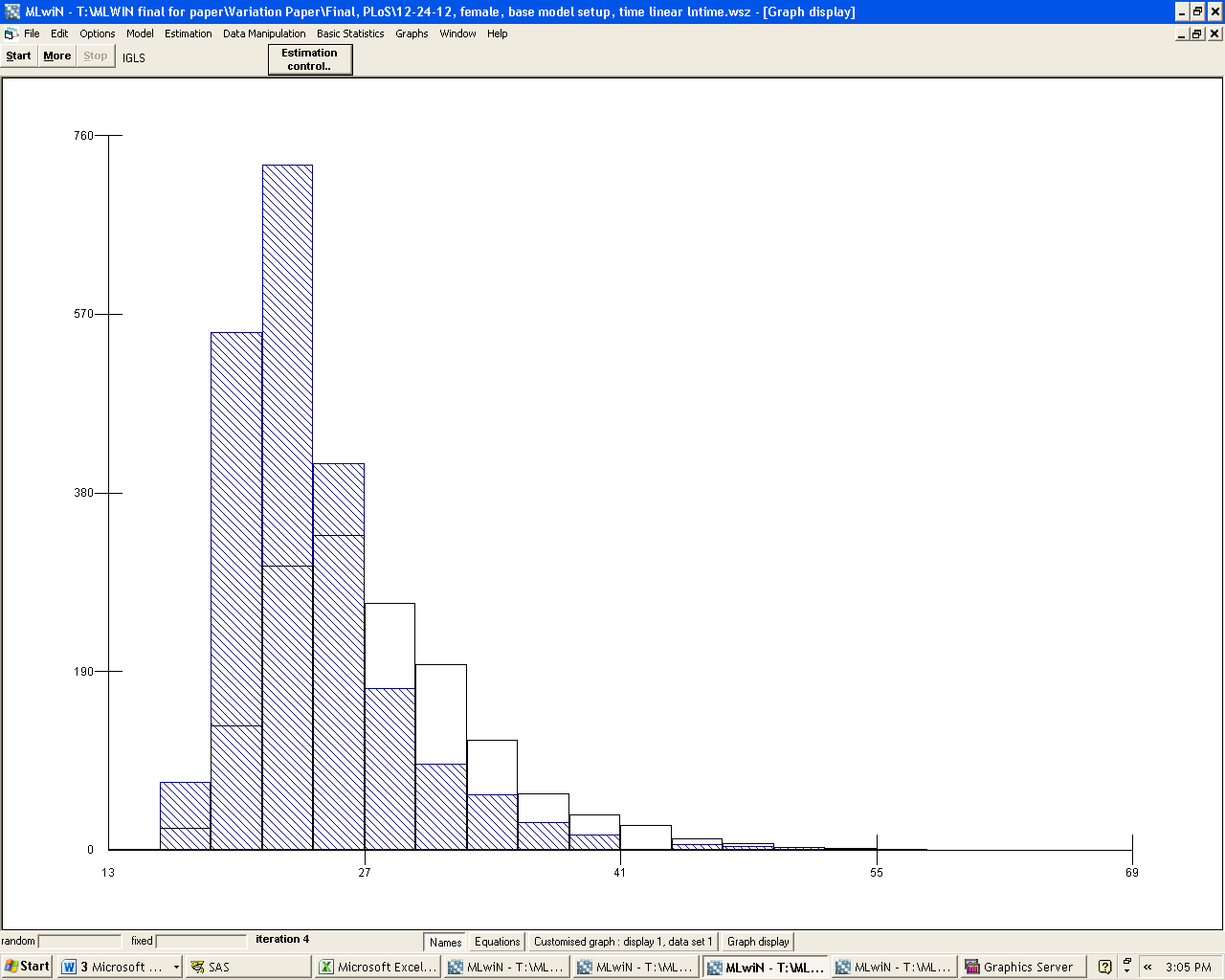
**

**B**


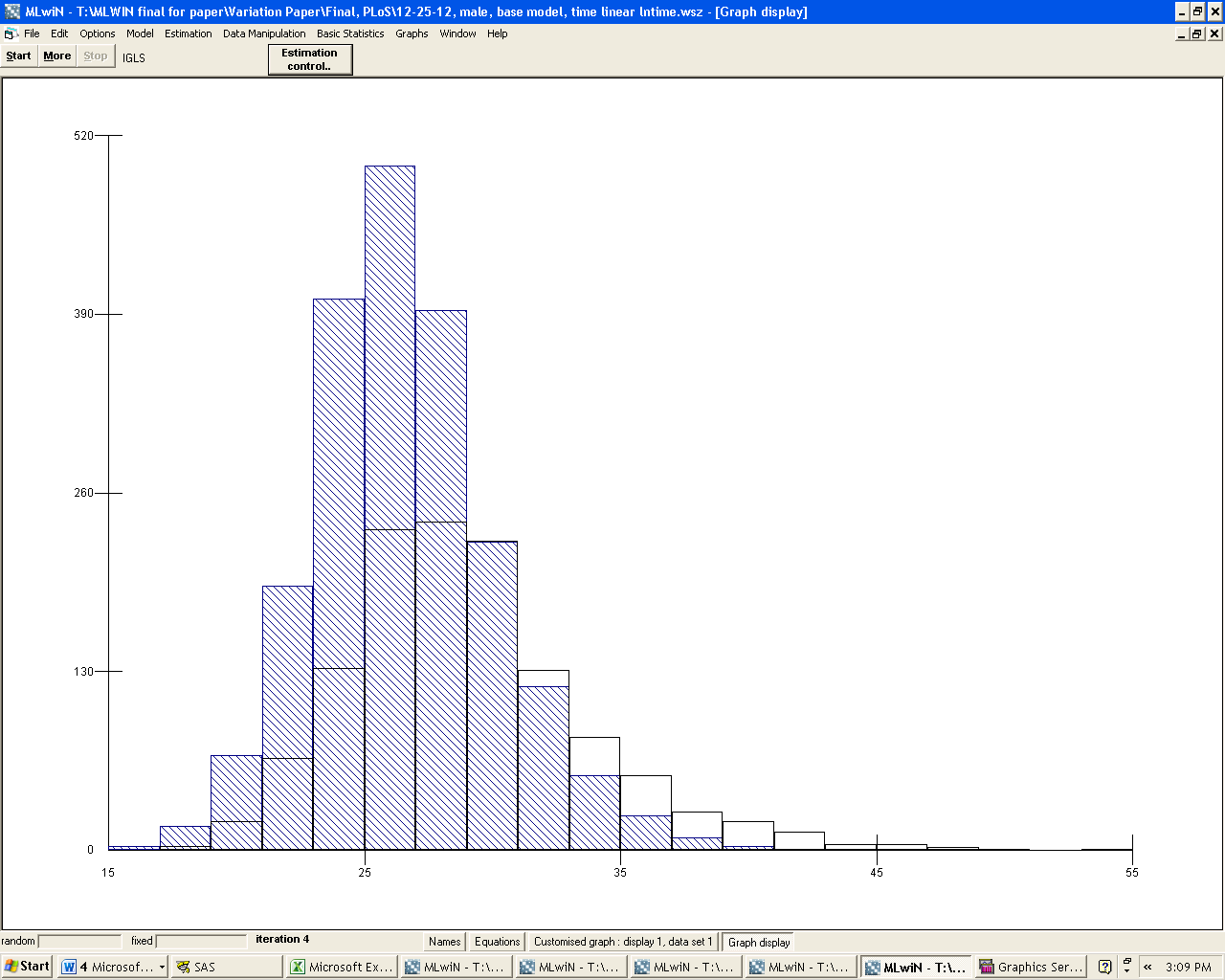


**Methods Note S1 in Appendix S1**

We used random effects models for these analyses rather than fixed effects models because of the greater efficiency in estimation. In the case of our data for women, a model that included fixed effects for individual would estimate regression parameters for 2366 women using 2365 parameters (a baseline level being set to 0). In contrast, a random effects model will only estimate one parameter for the individual level (the variance component), enabling more efficient and stable models. Also, because the random effect models examine how much each individual deviates from the mean BMI across individuals and then pools results into one variance estimate, results correspond to estimates for the entire population, assuming that the individuals in our study are representative of the population at large.

In the following we describe the basic longitudinal model used to relate an individual’s BMI at time *t* to their covariates (time varying and time-invariant) and individual-specific random effects for the intercept, linear time, and log-time. Using the subscripts *i* and *t* to denote individual and wave, respectively, we define $Y_{it}$ and $\mathbf{X}_{it}$ to be random variables representing BMI and a vector of *p* covariates for individual $i=1,\ldots,n_{I}$ at wave *t*=1,…,8. The constant 1 is the first element of $\mathbf{X}_{it}$. Let $\mathbf{\theta}_{i}=(\theta_{i1},\ldots,\theta_{i8})^{T}$, where $\theta_{it}$ is the individual-specific effect at wave *t*. The general form of the models used for our analysis is then:

$Y_{it}|\mathbf{\theta}_{i},\mathbf{X}_{it}\sim\text{Normal}(\theta_{i0}+\theta_{i1}t+\theta_{i2}\log(t)+\mathbf{\beta}^{T}\mathbf{X}_{it},\sigma^{2})$ (1)

$\mathbf{\theta}_{i}\sim\text{Normal}\left(\mathbf{0},\mathbf{\Sigma}\right)$, (2)

where $\mathbf{\beta}$ is a vector of *p* regression coefficients of the covariates, $\sigma^{2}$ is the variance of the random error associated with each observation (the idiosyncratic variation within an individual), and$\mathbf{\Sigma}$ is the population covariance matrix of the individual-specific effects $\mathbf{\theta}_{i}=(\theta_{i0},\theta_{i1},\theta_{i2})^{T}$. The model has a similar form to a small area estimation model at the individual-level. The term covariate $\mathbf{X}_{it}$ includes all individual- and neighborhood covariates as well as additional variables for linear time and the natural log of time.

Various versions of the model in (1) – (2) are estimated (e.g., those that stratify by Wave 1 BMI groups or condition on Wave 1 BMI). In addition, we also fit a related series of models that include a random effect for neighborhood and which are stratified by wave. Therefore, the model has the form of the model in (1) but replaces the three random effects for individual with single a random intercept for neighborhood and re-defining the indices *it* to be *hi*, where *h* denotes neighborhood.

We fitted all models using Markov Chain Monte Carlo (MCMC) methods to generate multiple iterative samples from the joint posterior distribution of the parameters, from which parameter estimates could be constructed. For prior distributions we use standard diffuse (i.e., minimal information) priors including flat priors for regression parameters and independent inverse-Gamma priors for variance components. We used generalized least squares models to generate starting values from which to run the MCMC models. We used 100,000 MCMC iterations to generate final estimates for each model with 10,000 iterations for the burn-in period that were not used in computing the estimates.
